# Cardiac and Systemic Thrombus Caused by Drug Abuse

**DOI:** 10.1155/2019/5083624

**Published:** 2019-04-07

**Authors:** John Eliveha, Shravani Vindhyal, Mohinder Vindhyal

**Affiliations:** Department of Internal Medicine, University of Kansas, 1001 N Minneapolis St., Wichita, KS 67214, USA

## Abstract

Drug abuse is an increasing concern all over the world especially in the United States. Methamphetamine have been well established to cause elevated body temperature, irregular heartbeat, seizures, and heart disease. We present a case of ventricular thrombus with systemic emboli in a patient with dilated cardiomyopathy after methamphetamine use.

## 1. Introduction

Methamphetamine is a sympathomimetic amine that increases synaptic concentration of serotonin, noradrenaline, and dopamine, creating hallucinogenic, euphoric, and stimulant effects [1]. Cardiovascular complications are the second leading cause of death in methamphetamine users [2]. The cardiac complications of methamphetamines are hypothesized from a variety of mechanisms such as hypertension, tachycardia, and myocardial toxicity with cellular death, fibrosis, and contraction band necrosis [3, 4].

## 2. Case Presentation

**Figure 1:**
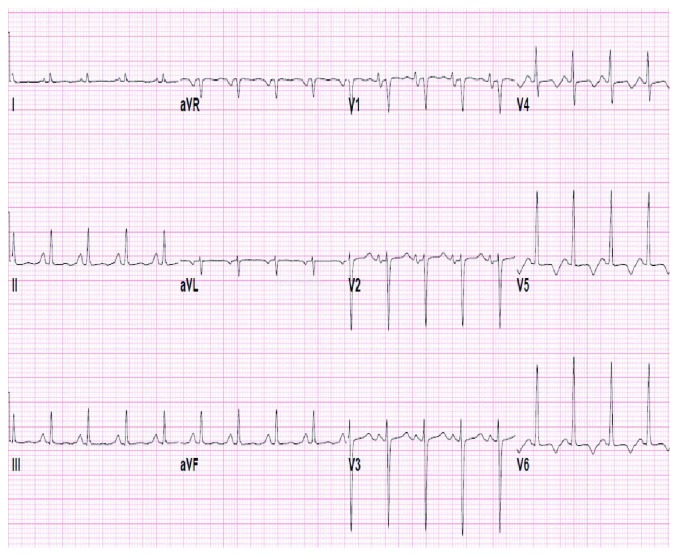
EKG showing nonspecific ST-T wave changes.

**Figure 2:**
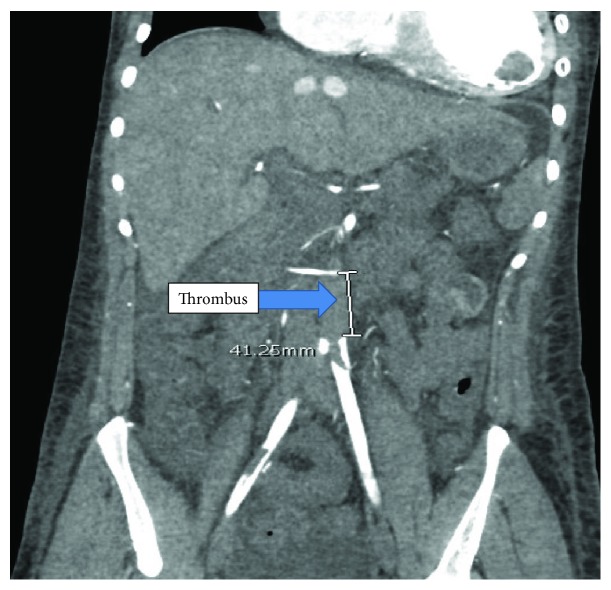
CT angiogram showing a massive AAA thrombus beyond the renal arteries.

**Figure 3:**
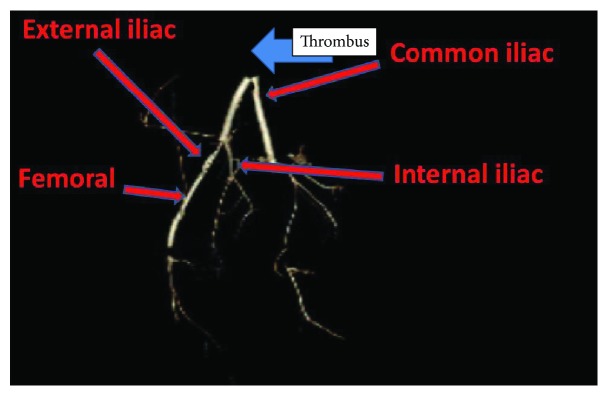
CT angiogram reconstruct showing large segmental aortic occlusions.

**Figure 4:**
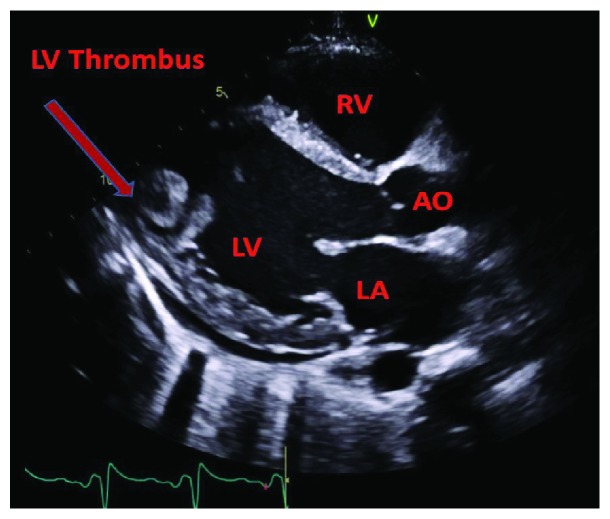
Parasternal long axis view showing LV thrombus. LA: left atrium; AO: aorta; RV: right ventricle; LV: left ventricle.

**Figure 5:**
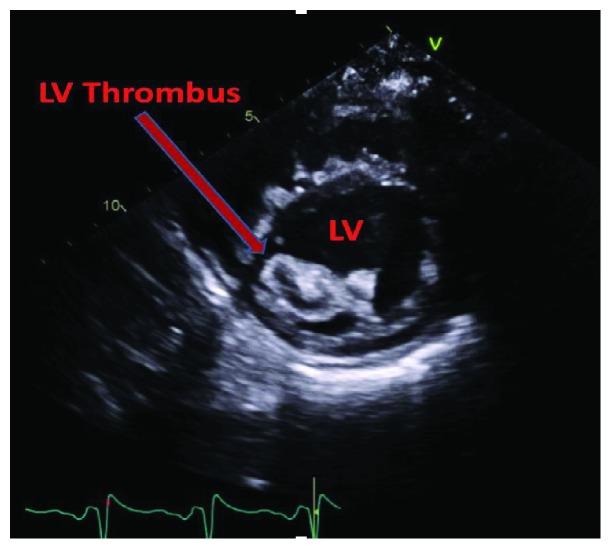
Parasternal short axis view. Mid to apical segment showing LV thrombus. LV: left ventricle.

**Figure 6:**
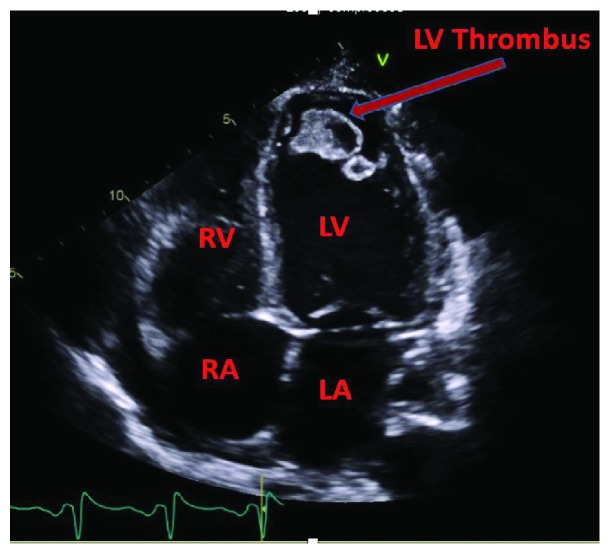
Apical four-chamber view showing LV thrombus. LA: left atrium; RA: right atrium; RV: right ventricle; LV: left ventricle.

A 24-year-old female with a history of HIV/AIDS, nonischemic cardiomyopathy, and methamphetamine and marijuana abuse presented with acute onset lower extremity pain. The patient reported to have snorted methamphetamine overnight and woke up with severe lower extremity pain as well as inability to move. Surgical history was significant for excision of sublingual glands. Family history: mother was HIV positive; both mother and father had significant history of substance abuse. She drank one to two alcoholic drinks per week and was an everyday smoker, between 1/4 pack and 1/2 pack per day. Her vital signs were significant for tachycardia, tachypnea, and hypotension. Lower extremity examination was positive for tender lower extremities with no palpable dorsalis pedis, posterior tibial, and popliteal pulses bilaterally. Initial lab tests were significant for lactic acidosis, acute kidney injury, EKG with nonspecific ST-T wave changes (Figure 1), elevated troponin, 1.08 ng/ml, peaked at 3.5 ng/ml and urine drug screen was positive for methamphetamine and marijuana. Hemoglobin 11.9 gm/dl, white blood cells 10.4, platelets 178, sodium 139 mmol/liter, potassium 4.1 mmol/liter, chloride 104 mmol/liter, bicarbonate 20 mmol/liter, BUN 11 mg/dl, creatinine 1.3 mg/liter, blood glucose 141 mg/dl, AST 68 units/liter, ALT 41 units/liter, total bilirubin 0.9 grams/dl, alkaline phosphatase 107 IU/liter, and INR 1.8. Arterial and venous duplex of the lower extremities revealed no blood flow. CT angiogram showed large segment aortic occlusion (4 cm) just beyond the renal arteries and partial occlusive thrombus in the superior mesenteric artery with early ischemia (Figures 2 and 3). There were also multiple areas of bilateral renal infarcts left greater than right with the main renal arteries patent bilaterally. Transthoracic echocardiogram showed an echo dense mass, 2.4 cm × 2.8 cm, 1.2 cm × 2.0 cm in size, in the left ventricle with defined margins that are distinct from the endocardium seen throughout systole and diastole, consistent with left ventricular thrombus (Figures 4–6). Ejection fraction was estimated to be 15 %, with increased wall thickness and grade 3 diastolic dysfunction. There was mild to moderate mitral and tricuspid regurgitation with normal valve structure. CT head was obtained due to an altered mental status which was negative for acute bleeding. The patient became profoundly hypotensive which was likely due to cardiogenic shock despite being on maximum vasopressors. She developed limb ischemia due to compartment syndrome requiring fasciotomy. Interventional radiology and vascular surgery were consulted, surgical thrombectomy was done. The patient's status worsened developing rhabdomyolysis, shock liver, and acute kidney injury with severe metabolic acidosis. She could not tolerate continuous renal replacement therapy. The patient had an episode of ventricular fibrillation and expired after three days of being hospitalized.

## 3. Discussion

Methamphetamine cause myocardial damage via multifactorial mechanisms. Excessive catecholamine activity is thought to be the primary mode underlying the cardiotoxic effects of methamphetamines, leading to excessive vasoconstriction, vasospasm, rapid heart rate, and high blood pressure [5]. Tachycardia leads to myocardial necrosis; increased blood pressure leads to fibrosis; vasospasm leads to hypertrophy of cells. Direct cardiac toxicity including hypertrophy and other damage in the heart has been well established, but the understanding of the pathophysiology is unknown [5].

A major complication of acute myocardial infarction is left ventricular thrombus formation. Risk factors for thrombus formation include large infarct size (EF < 30%), severe apical akinesia or dyskinesis, formation of ventricular aneurysms, and anterior myocardial infarction [6]. Virchow's triad is a prerequisite for in vivo thrombus formation. LV wall regional akinesia and dyskinesia result in stasis. Prolonged ischemia leads to subendocardial tissue injury with inflammatory changes. Acute MI patients have increased concentrations of prothrombin, fibrinopeptide A, and von Willebrand factor and decreased concentrations of ADAMTS 13 [7]. Left ventricular thrombus can occur within 24 hours of MI [8]. Patients can present with left ventricular thrombus after unrecognized myocardial infarction. Conditions that increase the risk for systemic embolization include severe congestive heart failure, diffuse left ventricular dilation, systolic dysfunction, previous embolization, advanced age, and the presence of left ventricular protruding or mobile thrombus [7].

Our patient had a prolonged history of methamphetamine abuse. She had chronic heart failure with reduced ejection fraction, 10-15% with severe dilated cardiomyopathy and diffuse hypokinesis. We hypothesized that catecholamine storm due to acute methamphetamine intoxication caused acute myocardial infarction. This was unrecognized mainly because of atypical presentation. Severe dyskinesia along with diffuse wall motion abnormalities led to stasis of blood and eventual thrombus formation. The patient had significant risk factors for systemic embolization including severe congestive heart failure and systolic dysfunction, leading to systemic embolization.

A high index of suspicion is paramount to making a diagnosis. Presence of risk factors, especially presence of severely reduced ejection fraction without anticoagulation, should prompt physicians to look out for left ventricular thrombus. Diagnosis can be made using two-dimensional transthoracic echocardiogram which is cheap, is readily available, and has excellent specificity, 85-90%, and sensitivity of 95% in detecting left ventricular thrombus [9]. Cardiac magnetic resonance imaging (CMR) with contrast delayed enhancement (DE) has significantly better accuracy than TTE for the diagnosis of LV thrombus, 99% specificity, and 88% sensitivity [9, 10]. DE-CMR is nowadays considered the gold standard for diagnosis. Vitamin K antagonist is the standard of treatment. The European guidelines recommend antivitamin K treatment for 3-6 months while the American guidelines recommend indefinite treatment for patients with low risk of bleeding [7].

## 4. Conclusion

Physicians should be aware of the cardiac complications of methamphetamine. Left ventricular thrombus with systemic emboli is a potential complication of chronic methamphetamine use.

Usually, patients with decreased ejection fraction are at a higher risk.
